# Relationships between Workload, Heart Rate Variability, and Performance in a Recreational Endurance Runner

**DOI:** 10.3390/jfmk6010030

**Published:** 2021-03-22

**Authors:** Daniel Boullosa, André R. Medeiros, Andrew A. Flatt, Michael R. Esco, Fabio Y. Nakamura, Carl Foster

**Affiliations:** 1 INISA, Federal University of Mato Grosso do Sul, Campo Grande 79070-900, Mato Grosso do Sul, Brazil; 2 Federal District Education Secretary, Brasilia 72302-505, Brazil; mdt.ricarte@hotmail.com; 3 Biodynamics Laboratory, Department of Health Sciences, Armstrong State University, Savannah, GA 31419, USA; aflatt@georgiasouthern.edu; 4 Exercise Physiology Laboratory, Department of Kinesiology, University of Alabama, Tuscaloosa, AL 870312, USA; mresco@ua.edu; 5 Research Center in Sports Sciences, Health Sciences and Human Development, CIDESD, University Institute of Maia, ISMAI, 4475-690 Maia, Portugal; fabioy_nakamura@yahoo.com.br; 6 Department of Exercise and Sports Science, University of Wisconsin-La Crosse, Lacrosse, WI 54601, USA; cfoster@uwlax.edu

**Keywords:** autonomic control of HR, vagal modulations, vagal-sympathetic effect, training monitoring, endurance performance

## Abstract

Background: The association between heart rate variability (HRV), training load (TL), and performance is poorly understood. Methods: A middle-aged recreational female runner was monitored during a competitive 20-wk macrocycle divided into first (M1) and second mesocycle (M2) in which best performances over 10 km and 21 km were recorded. Volume (km), session rating of perceived exertion (sRPE), TL, and monotony (mean TL/SD TL) were the workload parameters recorded. The root mean square of the successive differences in R-R intervals (RMSSD), its coefficient of variation (RMSSDcv), and the RMSSD:RR ratio were the HRV parameters monitored. Results: During M2, RMSSD (*p* = 0.006) and RMSSD:RR (*p* = 0.002) were significantly increased, while RR was significantly reduced (*p* = 0.017). Significant correlations were identified between monotony and volume (*r =* 0.552; *p* = 0.012), RR (*r =* 0.447; *p* = 0.048), and RMSSD:RR (*r =* −0.458; *p* = 0.042). A sudden reduction in RMSSD (from 40.31 to 24.34 ms) was observed the day before the first symptoms of an influenza. Conclusions: The current results confirm the practicality of concurrent HRV and sRPE monitoring in recreational runners, with the RMSSD:RR ratio indicative of specific adaptations. Excessive training volume may be associated to both elevated monotony and reduced RMSSD:RR. Identification of mesocycle patterns is recommended for better individualization of the periodization used.

## 1. Introduction

Heart rate variability (HRV) is a valid and accessible cardiac-autonomic marker that has been promoted as a technique for monitoring training of recreational runners [1]. The objectives of routine assessment of HRV among athletes include selecting long-term training methods [2], modifying daily exercise prescriptive factors [3], and identifying positive and negative adaptations [4]. There are numerous HRV indices. The square root of mean squared difference of successive R–R intervals (RMSSD) is a robust index of vagal autonomic function that is commonly employed by recreational runners [4]. Indeed, its coefficient of variation (RMSSDcv) and the RMSSD:RR ratio are simple HRV parameters that have been proposed to identify individual responses to endurance training [5,6]. The RMSSD:RR ratio has been proposed to identify vagal saturation as it normalizes vagal modulations by the RR intervals, therefore relating vagal and sympathetic modulations [7].

The associations of endurance running performance with autonomic adaptations and with markers of internal and external training loads separately are well documented. However, there is only one study [8] that has documented a dose–response relationship between heart rate (HR)-derived training impulse (TRIMP), power spectral analyses of HRV, and marathon times. These results suggest a possible sympathetic drift towards the end of the preparation period that positively correlated with performance. However, the weekly autonomic changes associated with weekly training workload indices were not reported in this previous study [8]. This information would be of interest given the previous suggestions on the existence of individual autonomic profiles associated with training cycles and periodization in other endurance sports [5,7]. For instance, a simultaneous reduction in LnRMSSD and LnRMSSD:RR during the final week preceding competition appeared to be indicative of optimal performance in an elite triathlete [7]. In another study with elite rowers [5], different autonomic adaptations to training at different time points of the preparation were also observed. Therefore, identification of how weekly training workload relates to autonomic status would help to better manage training load.

Thus, we present a case report of a recreational female runner who completed a 20-wk competitive macrocycle. Daily HRV and training indices were recorded for subsequently identifying associations between HRV, training workload indices, and running performances. Based on previous studies, we would expect reduced RMSSD and RMSSD:RR prior to better performances.

## 2. Materials and Methods

The recreational runner is a middle-aged female (50 years; 1.59 m; 50–52 kg; maximum oxygen consumption [VO_2_max] = 56 mL·kg^−1^·min^−1^) with more than 10 years of endurance training experience, first as a triathlete and more recently (last 7 years) as a road runner. The training history and periodization used have been described elsewhere [9]. Briefly, she completed a 20-week competitive macrocycle, after a 3-month preparatory mesocycle, in which she competed in two 10-km and three 21-km road races in the city of Brasilia, under thermoneutral environmental conditions (<23 °C and <50% relative humidity). Briefly, all the races were completed in early (7:00 a.m.) morning, with similar profiles. Best performances in both distances occurred in the second part of the competitive macrocycle, achieving 99.3% and 97.8% of her best performances for 10 km and 21 km, respectively (recorded 6 years before). Therefore, the competitive macrocycle was divided into first mesocycle (M1 = 10 first weeks) and second mesocycle (M2 = 10 last weeks). She gave her consent for the public use of her data for this case study.

Workload indices included session Rating of Perceived Exertion (sRPE) and its derived indices of training load (sRPE × time in minutes) and monotony (mean weekly training load/SD of weekly training load) [10]. Daily volume in km was recorded with a GPS unit (Forerunner 630, Garmin, Olathe, KS, USA). The typical weekly microcycle (5–7 sessions) included two strength-training sessions plus 20–30 min of submaximal uphill runs on treadmill; one to two running sessions of ‘cruise intervals’ (at or slightly below the competitive pace), and some intervals at maximum aerobic speed (MAS) in the weeks before competitions. On designated recovery days, she performed 1–2 easy short runs or runs plus walks of 30–60 min, interspersed with some maximum speed progressions over 100-m; and a single long easy run of 70–100 min. The training intensity distribution was “polarized” (75-80/5/15-20) as previously documented [9].

RR intervals were recorded for 2 min (after 1 min of stabilization) every morning, in supine position after awakening, with a validated HR strap (H7, Polar Electro Oy, Kempele, Finland), and exported via Bluetooth to a mobile App (Elite HRV, Asheville, NC, USA). The RR and RMSSD values obtained with the mobile App were subsequently recorded and exported to a custom Excel^®^ spreadsheet, in which weekly RMSSDcv (i.e., [SD of RMSSD/mean RMSSD] × 100) and RMSSD:RR (i.e., mean RMSSD/mean RR) were calculated.

Values are presented as mean ± SD. After normality distribution confirmation, differences between weekly HRV indices in M1 and M2 were performed with a non-paired t test, and effect size (ES) via a Cohen’s d. The smallest worthwhile change (SWC) was also calculated as 0.3 × SD of week 1 [11]. The relationships between training workload and HRV indices were performed with a Pearson product correlation coefficient (*r*). Statistical significance was set at 5%.

## 3. Results

The evolution of training workload, HRV indices, and running performances (10-km and 21-km running times) over the 20-week macrocycle are presented in Table 1. Differences between M1 and M2 for dependent variables are presented in Table 2. Of note, a sudden reduction in RMSSD (from 40.31 to 24.34 ms) during week 8 was observed the day before the first symptoms of an influenza, which was followed by 2 days of disrupted training.

**Figure 1:**
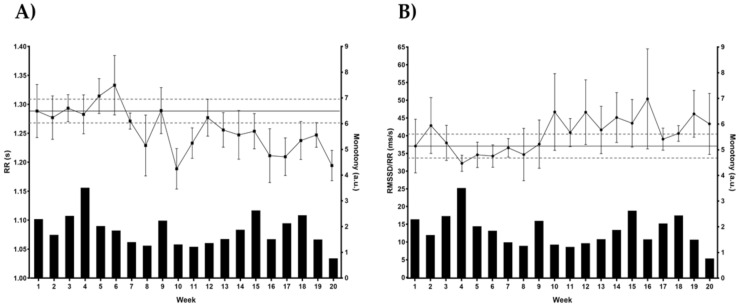
Concurrent weekly changes of monotony and RR intervals (**A**), and monotony and the RMSSD:RR ratio (**B**). The black dots represent the HRV parameters, while the black bars represent the monotony scores.

There were significant correlations between the km completed each week with training load (*r =* 0.738; *p* < 0.00) and monotony (*r =* 0.552; *p* = 0.012). Conversely, some HRV indices were correlated among them. RMSSD:RR correlated with RMSSD (*r =* 0.973; *p* = 0.000), RMSSDcv (*r =* 0.526; *p* = 0.027), and RR (*r =* −0.581; *p* = 0.007), while RMSSD correlated with RMSSDcv (*r =* 0.499; *p* = 0.025). Further, monotony was correlated with weekly HRV indices: RR (*r =* 0.447; *p* = 0.048) and RMSSD:RR (*r =* −0.458; *p* = 0.042). There were no correlations between training load and HRV parameters. Concurrent weekly changes of monotony and RR, and monotony and RMSSD:RR are shown in Figure 1.

## 4. Discussion

The main and novel observation of this case report was the association between monotony, an index of weekly load periodization, and both RR and RMSSD:RR. In addition, our hypothesis was partially confirmed with only reduced RR in M2 being indicative of improved performances. However, a greater RMSSD:RR was observed with better performances in M2, which was strongly correlated to greater RMSSD values in this mesocycle. Thus, simultaneous enhancement of vagal (↑RMSSD) and, probably sympathetic (↓RR and ↑RMSSD:RR) modulations would be suggestive of better adaptations and thus improved performances. Furthermore, these autonomic adaptations would be related to reduced monotony scores. Therefore, simultaneous recording of weekly monotony and RMSSD:RR may be important monitoring tools for endurance runners and other endurance athletes.

The results are in alignment with one study with an elite triathlete [7], suggesting an increased RMSSD associated with positive adaptations and reduced RMSSD:RR associated with improved performances. However, another study with world-champion rowers [5] exhibited consistent substantial reductions in RMSSD:RR prior to outstanding performances. Meanwhile, we did not observe the relationship between reduced RMSSDcv and improved performances observed in another case study with a male recreational athlete [6]. Differences between studies may be attributed to differences between HRV recording characteristics (i.e., position and duration of recordings), periodizations used, and sports demands. However, the increased RMSSD:RR, which would be associated to enhanced vagal modulations and also sympathetic activity, is consistent with previous reports of enhanced performances in samples of recreational runners with greater RMSSD values [4,6], and a possibly increased sympathetic activity near competition [8]. This reinforces the value of the RMSSD:RR ratio to monitor recreational endurance runners, with RR intervals (i.e., a vagal-sympathetic effect index) [12] being a complementary HRV parameter to the most used RMSSD by practitioners. Further studies with samples of recreational runners are needed to corroborate these observations.

The most novel and important observations were the associations identified between monotony with RR (*r =* 0.447; *p* = 0.048) and RMSSD:RR (*r =* −0.458; *p* = 0.042). These correlations are contrary to the desired autonomic adaptations, thus confirming the well-known negative effects of monotony on health and performance of athletes [10]. Monotony was also associated with weekly volume (*r =* 0.552; *p* = 0.012) which, in turn, was associated with training load (*r =* 0.738; *p* < 0.00). This would suggest that volume, a well-known pre-requisite for endurance adaptations, could also favor negative adaptations when associated with high monotony. Of note, the association between training volume and monotony would be mathematically expected in most cases. However, there were no significant differences between training workload parameters between mesocycles, although the ES revealed M1 as the most demanding mesocycle (see Table 2). In this regard, a more detailed analysis reveals that the best performance occurred in week 12 (39:56 in 10 km, which represents 99.3% of her best) with the peak volume achieved, in this case, 3 weeks before competitions instead of 2 weeks as for the other races. Further, this peak volume (i.e., 82 km) was associated to very low monotony scores (i.e., 1.32–1.22) during the 2 weeks of tapering before competition. Therefore, identification of weekly volumes should be accompanied by examination of monotony scores over several weeks, and no single weeks, to identify individual patterns to be replicated in future periodizations. This is an important consideration given the well-known limitation of periodizations to induce peak performances in a purported time. These observations also reinforce the risk associated to high volumes, which are very typical of recreational runners training for performance purposes [1]. In this regard, the approach of the current case report agrees with the recent suggestions on the need for combining both internal and external training load indices for optimized training load monitoring in runners [13]. Therefore, the concurrent use of sRPE and HRV would expand the validity of monotony scores for training monitoring [14], which should be confirmed in further studies.

One interesting finding was the sudden reduction of vagal modulations (i.e., RMSSD = 24.34) because of an influenza. Interestingly, this sudden reduction occurred 1 day before any symptom and served to cancel the programmed training on that day. After 2 days of rest, the runner returned progressively to normal training without any relevant issue to be reported. This is a relevant observation that should be considered by runners exposed to any viral infection (e.g., SARS-CoV), with further studies needed to confirm these observations.

This case study is not without limitations. As this is a single case report, generalization of these results should be considered with caution. Of note, specific characteristics of training and daily activities of the runner may be related to our observations. For instance, the runner followed an “Evolutionary periodization” [9], which accounts for management of both training loads and lifestyle habits, including professional activities, sleeping routines [15], nutritional strategies [16], and incidental physical activity [17] among others. In this regard, as the pre-planned loads were adapted on a daily basis, with consideration of all these factors (including HRV morning data [18]), we do not know if a fixed periodization would result in similar outcomes. In addition, these associations may be different when using other HRV protocols [19], parameters [20], and Apps with different correction algorithms [21,22,23]. 

## 5. Conclusions and Practical Applications 

We identified specific autonomic adaptations related to training workload parameters and better performances in a middle-aged recreational female runner. Specifically, an enhanced RMSSD:RR was associated to reduced monotony, a consistent response during the mesocycle of best performances. In addition, a reduction in vagal modulations during the first days of an influenza was also observed. Future studies with runners of different age, sex and levels should confirm these important observations.

Following the current observations, it may be recommended to daily record sRPE, RMSSD, and RR. The subsequent calculation of training load (i.e., sRPE × time), monotony (i.e., mean weekly training load/SD of weekly training load), and RMSSD:RR, would therefore assist to monitor changes of these parameters on a weekly and mesocycle basis, with respect to changes in training volume (km) and running performance.

## Figures and Tables

**Table 1:** Weekly mean values for HRV, training workload parameters, and competitive performances.

Week	Distance (km)	Training Load (sRPE × Time)	Monotony	RMSSD (ms)	RMSSDcv (%)	RR (s)	RMSSD/RR (ms/s)	Running Performances
1	68	344	2.29	47.75	34.82	1.29	37.06	
2	55	275	1.68	54.73	34.22	1.28	42.85	
3	75	478	2.42	49.09	21.73	1.29	37.95	
4	83	512	3.51	41.29	14.74	1.28	32.19	
5	77	473	2.02	45.46	17.53	1.31	34.59	
6	65	299	1.85	45.64	20.36	1.33	34.23	41:58 (10-km)
7	77	623	1.40	46.47	12.63	1.27	36.56	
8	64	462	1.26	42.60	40.70	1.23	34.66	
9	82	509	2.23	48.48	34.19	1.29	37.60	
10	65	319	1.31	55.48	39.73	1.19	46.67	1:28:25 (21-km)
11	53	262	1.22	50.39	15.26	1.23	40.87	
12	55	381	1.36	59.52	34.60	1.28	46.61	39:56 (10-km)
13	52	363	1.52	52.26	28.21	1.26	41.61	
14	93	531	1.88	56.27	27.12	1.25	45.11	
15	70	522	2.63	54.53	25.64	1.25	43.49	
16	63	395	1.51	61.02	52.34	1.21	50.37	1:26:45 (21-km)
17	55	257	2.13	47.19	16.22	1.21	39.02	
18	84	560	2.44	50.27	12.37	1.24	40.62	
19	47	400	1.50	57.55	23.94	1.25	46.15	
20	61	212	0.77	51.73	33.42	1.19	43.31	1:26:33 (21-km)

**Table 2:** Comparison of HRV and training workload parameters between M1 and M2.

	M1	M2	*t*-Test (*p*)	Cohen’s d
RMSSD (ms)	47.7 (4.60)	54.1 (4.46)	0.006	−1.407
RMSSDcv (%)	27.1 (10.7)	26.9 (11.6)	0.976	0.014
RR (s)	1.28 (0.041)	1.24 (0.025)	0.017	1.207
RMSSD/RR (ms/s)	37.44 (4.34)	43.72 (3.41)	0.002	−1.620
Distance (km)	71.1 (9.06)	63.3 (14.90)	0.174	0.651
Training load (a.u.)	429 (114)	388 (121)	0.444	0.350
Monotony (a.u.)	2.00 (0.67)	1.69 (0.57)	0.296	0.483

## Data Availability

The data that support the findings of this study are available from the corresponding author upon reasonable request.
